# Histone Hyperacetylation as a Response to Global Brain Ischemia Associated with Hyperhomocysteinemia in Rats

**DOI:** 10.3390/ijms19103147

**Published:** 2018-10-12

**Authors:** Barbara Tóthová, Mária Kovalská, Dagmar Kalenská, Anna Tomašcová, Ján Lehotský

**Affiliations:** 1 Department of Molecular Medicine, Biomedical Center Martin, Jessenius Faculty of Medicine, Comenius University in Bratislava, 03601 Martin, Slovakia; tothova@jfmed.uniba.sk; 2 Department of Histology and Embryology, Jessenius Faculty of Medicine, Comenius University in Bratislava, 03601 Martin, Slovakia; kovalska@jfmed.uniba.sk; 3 Department of Medical Biochemistry, Jessenius Faculty of Medicine, Comenius University in Bratislava, 03601 Martin, Slovakia; kalenska@jfmed.uniba.sk (D.K.); benova111@uniba.sk (A.T.); 4 Department of Neuroscience, Biomedical Center Martin, Jessenius Faculty of Medicine, Comenius University in Bratislava, 03601 Martin, Slovakia

**Keywords:** homocysteine (Hcy), histone acetylation, global forebrain ischemia, ischemia-reperfusion injury (IRI), cortex, rat

## Abstract

Epigenetic regulations play an important role in both normal and pathological conditions of an organism, and are influenced by various exogenous and endogenous factors. Hyperhomocysteinemia (hHcy), as a risk factor for several pathological conditions affecting the central nervous system, is supposed to alter the epigenetic signature of the given tissue, which therefore worsens the subsequent damage. To investigate the effect of hHcy in combination with ischemia-reperfusion injury (IRI) and histone acetylation, we used the hHcy animal model of global forebrain ischemia in rats. Cresyl violet staining showed massive neural disintegration in the M1 (primary motor cortex) region as well as in the CA1 (cornu ammonis 1) area of the hippocampus induced by IRI. Neural loss was significantly higher in the group with induced hHcy. Moreover, immunohistochemistry and Western blot analysis of the brain cortex showed prominent changes in the acetylation of histones H3 and H4, at lysine 9 and 12, respectively, as a result of IRI and induced hHcy. It seems that the differences in histone acetylation patterns in the cortical region have a preferred role in pathological processes induced by IRI associated with hHcy and could be considered in therapeutic strategies.

## 1. Introduction

The epigenetic mechanism of gene regulation in brain tissue has been intensively studied under various physiological and pathological conditions [1,2]. Epigenetic marks represent group of processes and structures that alter gene expression by regulating DNA accessibility. These traits are heritable and do not directly affect the DNA sequence. Epigenetic modifications including DNA methylation, posttranslational modification of histones or noncoding RNA interactions play a pivotal role in a plethora of physiological functions and, remarkably, in many disorders [3,4].

Aberrant regulation of homocysteine (Hcy) metabolism is a sulfur-containing amino acid derived from methionine that leads to the hyperhomocysteinemia (hHcy). In addition to the pleiotropic biochemical properties of Hcy, it can also act through epigenetic pathways by alteration in DNA methylation as a result of imbalance in the levels of biochemical components of Hcy cycle [5,6,7]. In the process of Hcy to methionine conversion S-adenosylmethionine (SAM) arises, a major methyl donor for all methylation reactions within the cell. SAM is further methylated to S-adenosylhomocysteine (SAH). The methylation potential of a cell can be defined by the SAM:SAH ratio [8,9], which is remarkably decreased in hHcy conditions induced both by the deficiency of vitamin B_6_ and B_12_ or due to genetic polymorphisms of metabolic genes [10]. Several studies support the evidence that hHcy can lead to global DNA hypomethylation [11,12,13,14]. Remarkably, hypomethylation is associated with reduced recruitment of histone deacetylases [15]. This in turn causes accumulation of acetylated H3 and H4 histones at lysine residues [16]. DNA hypomethylation and histone acetylation are associated with a transcriptionally permissive state of chromatin, although the open chromatin state is more available for protein molecules that suppress gene expression. Conversely, Hcy-induced DNA hypomethylation of gene promoter regions results in selective gene upregulation that contributes to augmented tissue injury [17,18].

HHcy is also identified as one of the risk factors and a target of preventive strategies for atherosclerosis, vascular disorders including cerebral stroke, as well as for neurodegenerative diseases and congenital defects [19,20,21]. Besides, these pathological conditions are associated with significant changes in the epigenetic state of the injured organs and the corresponding tissue. For instance, acute cerebral ischemic stroke is related to the increased DNA methylation accompanied with decreased acetylation of histones in neural tissue [22,23,24,25]. The reports on effects of hypoxia/ischemia upon levels of acetylated histones in hHcy conditions in various brain regions are still insufficient. An increasing body of evidence points to the imbalance in histone de/acetylation in different pathologies affecting the central nervous system [26]. Moreover, various gene regions are targeted by different patterns of acetylation resulting in multiple functional modifications. Acetylation of histone H3 at lysine 9 (H3K9ac) is presumably located in the regions surrounding the gene transcriptional start sites, whereas histone H4 lysine 12 acetylation (H4K12ac) is elevated in the promoter and transcribed regions of active genes [27]. This may suggest the different functions of each acetylation pattern. For instance, upregulation of H3K9ac leads to decreased expression of proinflammatory genes [28,29].

Cortical degeneration profoundly affects proper brain function and behavior. Several brain diseases selectively impact specific cortical regions, leaving others relatively intact. For example, Alzheimer’s disease (AD) as a severe neurodegenerative disorder, leads to functional deficit and cognitive decline caused by progressive cell loss in certain cortical systems. Among AD, brain cortex is affected in different ways also by other neurological conditions such as schizophrenia or dementia [30]. Our recent studies have shown that global IRI with combination of hHcy leads to more extensive neurodegeneration predominantly in the hippocampus, but to the morphological damage of cortical neurons as well [31,32], in spite of the various vulnerability of neurons to degeneration and apoptosis [33].

Until now, most studies have been focused to the epigenetic effect of Hcy, especially in the relationship with DNA or histone methylation, and not in the association with histone acetylation. Moreover, just a small number of these studies investigate the combination of cerebral IRI and hHcy condition in connection with histone acetylation. In the present study, we attempt to ascertain for the first time the effect of IRI in combination with induced hHcy to examine the extent of neuronal damage and the changes in acetylation level of histones H3 and H4 at specific lysine residues in vulnerable brain areas, such as rat cerebral cortex.

## 2. Results

### 2.1. Cresyl Violet Staining

**Figure 1:**
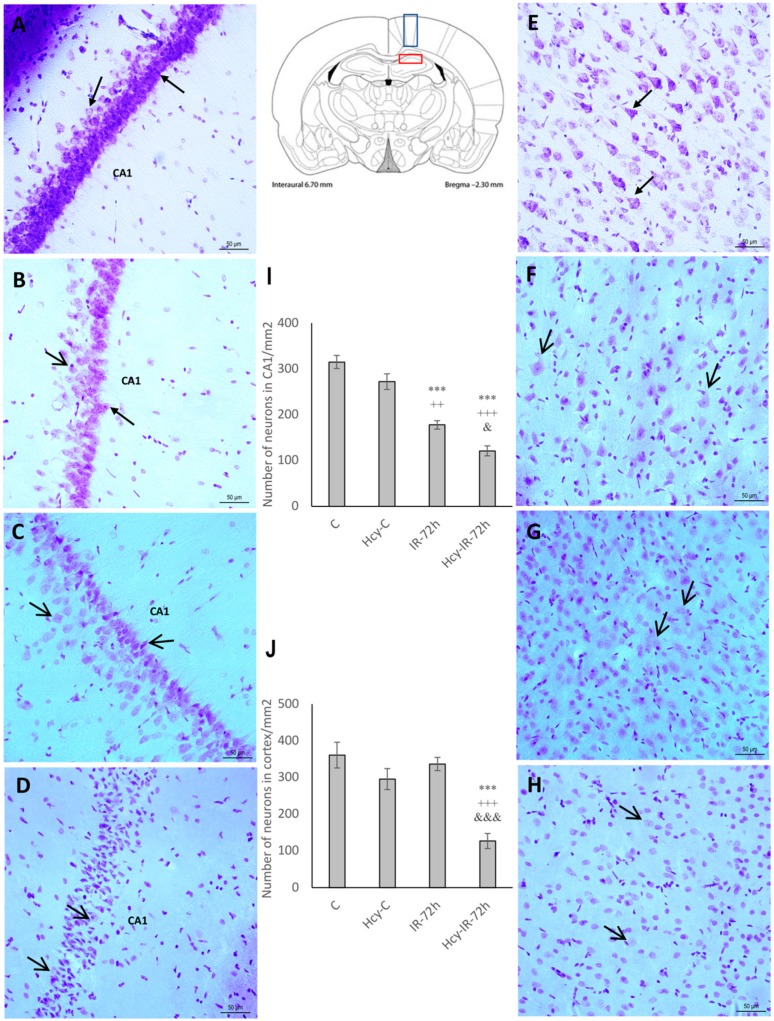
Cresyl violet stained rat brain sections and statistical evaluation of changes in number of vital neurons in the CA1 (cornu ammonis 1) region of hippocampus and M1 (primary motor cortex) region of rat brain cortex. Bright-field micrographs of CA1 region of hippocampus representing naive control (**A**), homocysteine-treated control (Hcy-C) (**B**), naive ischemia followed by 72 h of reperfusion (IR-72h) (**C**), Hcy-IR-72h (**D**) and micrographs of M1 region of rat brain cortex representing naive control (**E**), Hcy-C (**F**), naive IR-72h (**G**) and Hcy-IR-72h (**H**). Arrows indicate morphologically changed neurons, while arrow heads show vital neurons. Bar = 50 µm, *n* = 5/group. Schematic coronal rat brain section (according to Paxinos and Watson [34]) showing CA1 area of rat hippocampus (red rectangle) and M1 region (blue rectangle) of cerebral cortex. Number of vital neuronal cells in the CA1 area of hippocampus (**I**) and M1 region of rat brain cortex (**J**) in naive control, Hcy-C, naive IR-72h and Hcy-IR-72h. Results are presented as mean ± SEM, *n* = 5/group. *** *p* < 0.001 versus the control value; ^++^ *p* < 0.01 and ^+++^ *p* < 0.001 versus the control with Hcy; ^&^ *p* < 0.05 and ^&&&^ *p* < 0.001 versus the corresponding reperfusion period.

In order to assess the impact of hHcy in association with ischemia and ischemia-reperfusion injury (IRI) to the extent of neuronal damage, we used cresyl violet staining, which is commonly used to identify the neuronal structure in brain and specifically stains Nissl bodies. In the naive control group (C), neural cells in the CA1 and M1 region appeared round with pale stained nuclei and purple Nissl bodies (Figure 1A,E).

In the Hcy-C group we observed a diminished number of neural cells when compared to the naive control (C), although without notable statistical significance in both CA1 hippocampal and M1 cortical regions (Figure 1B,F). Conversely, neurons in these brain regions showed obvious morphological changes with signs of swelling and intracellular organelles damage in naive IR-72h group (Figure 1C,G). These changes were more remarkable in the CA1 hippocampal area where the number of intact neurons decreased significantly to 56% (177.5 ± 9.2; *p* < 0.001) when compared to the naive control (C) and to 65% in comparison with Hcy-C group (272 ± 17.1; *p* < 0.01). Neurons in the CA1 region of Hcy-IR-72h group demonstrated more eminent damage then the naive IR-72h group (Figure 1D). More specifically, we observed massive loss of neurons and total disintegration accompanied by cytoplasm shrinkage and vacuolization. The number of surviving neurons in the Hcy-IR-72h group declined to 38% (120.75 ± 10.9; *p* < 0.001) when compared to the naive control (C) and to 44% (*p* < 0.001) in comparison to Hcy-C group in CA1 hippocampal area. Moreover, the number of intact neurons was significantly decreased to 68% (*p* < 0.05) when compared to the naive IR-72h group (Figure 1I).

In the Hcy-IR-72h group, neural cells localized in the M1 cortical region showed similar signs of damage (Figure 1H). Compared to the naive control (C), the number of surviving neurons in this brain area declined to 35% (126.25 ± 20.5; *p* < 0.001) and to 43% (*p* < 0.001) in comparison with Hcy-C group. Furthermore, there was a significant decrease of 38% (*p* < 0.001) between the naive IR-72h and Hcy-IR-72h groups in the M1 cortical region (Figure 1J).

### 2.2. Immunohistochemical Detection of Acetylated Histone H3 (Lys9) and H4 (Lys12)

**Figure 2:**
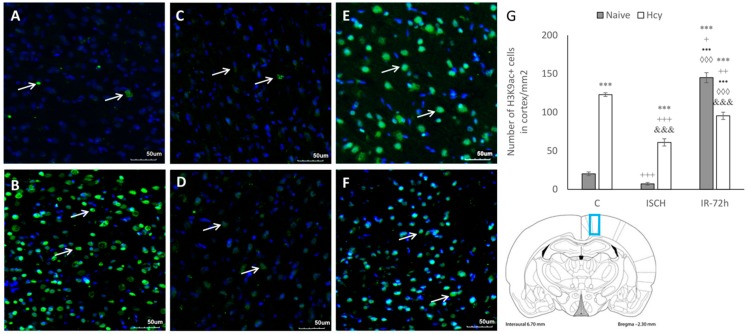
Fluorescence micrographs and statistical evaluation of H3K9ac positive (+) neurons in the M1 area (blue rectangle on the schematic coronal section) of rat brain cortex. Micrographs of naive control (**A**), Hcy-C (**B**), naive ischemic groups (ISCH) (**C**), Hcy-ISCH (**D**), naive IR-72h (**E**) and Hcy-IR-72h (**F**). Arrows indicate H3K9ac+ neuronal nuclei (green) in the M1 area of rat brain. Nuclei are co-stained with 4′,6-diamidino-2-phenylindole (DAPI) (blue). Bar = 50 µm, *n* = 5/group. (**G**) Number of H3K9ac+ nuclei in the M1 area in naive control, Hcy-C, naive ISCH, Hcy-ISCH, naive IR-72h and Hcy-IR-72h. Results are presented as mean ± SEM, *n* = 5/group. *** *p* < 0.001 versus the control value; ^+^ *p* < 0.05, ^++^ *p* < 0.01 and ^+++^ *p* < 0.001 versus the control with Hcy; ^•••^ *p* < 0.001 versus the naive ISCH group; ^◊◊◊^ *p* < 0.001 versus the ISCH with Hcy; and ^&&&^ *p* < 0.001 versus the corresponding reperfusion period.

**Figure 3:**
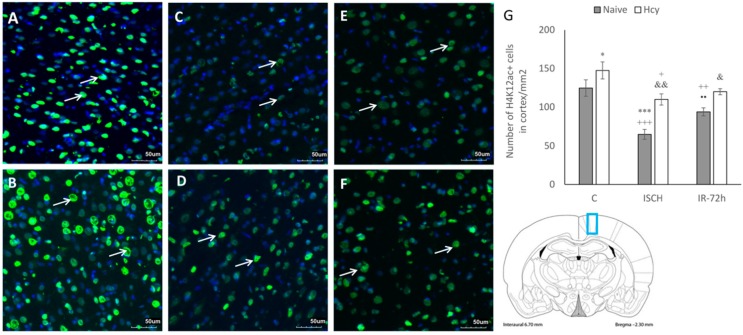
Fluorescence micrographs and statistical evaluation of H4K12ac positive (+) neurons in the M1 area (blue rectangle on the schematic coronal section) of rat brain cortex. Micrographs of naive control (**A**), Hcy-C (**B**), naive ISCH (**C**), Hcy-ISCH (**D**), naive IR-72h (**E**) and Hcy-IR-72h (**F**). Arrows indicate H4K12ac+ neuronal nuclei (green) in the M1 area of rat brain. Nuclei are co-stained with DAPI (blue). Bar = 50 µm, *n* = 5/group. (**G**) Number of H4K12ac+ nuclei in the M1 area in naive control, Hcy-C, naive ISCH, Hcy-ISCH, naive IR-72h and Hcy-IR-72h. Results are presented as mean ± SEM, *n* = 5/group. * *p* < 0.05 and *** *p* < 0.001 versus the control value; ^+^ *p* < 0.05, ^++^ *p* < 0.01 and ^+++^ *p* < 0.001 versus the control with Hcy; ^••^ *p* < 0.01 versus the naive ISCH group; ^&^ *p* < 0.05 and ^&&^ *p* < 0.01 versus the corresponding reperfusion period.

After IRI, the histological damage and possible histone posttranslational modifications are first detected and established in the hippocampus where they are transient, and later can also be detected in the cortex [1]. Therefore, as next we investigated the alterations in histone acetylation levels in M1 cortical region. Brain sections were subjected to immunohistochemical analysis to detect acetylated histone H3 at lysine 9 (H3K9ac) and histone H4 at lysine 12 (H4K12ac). The figures show representative pictures from the naive control (C) and Hcy-C group, ischemic groups (ISCH; Hcy-ISCH) and IRI groups with 72 h reperfusion (IR-72h; Hcy-IR-72h) (Figure 2 and Figure 3).

Signals corresponding to H3K9ac and H4K12ac were predominantly located within the nuclei. There were obvious changes in histone acetylation in all experimental conditions, with remarkable differences between the naive and Hcy-groups. As seen from the fluorescent micrographs, the lowest H3K9ac immunopositivity (+) was detected in the naive ISCH group and in the naive control (Figure 2A–F). Compared to the naive control (C), the number of H3K9ac immunopositive cells in naive ISCH group decreased, although not significantly, but highly increased to 716% (145 ± 6.5; *p* < 0.001) in the naive IR-72h group. The number of H3K9ac+ cells in the Hcy-C group was elevated to 607% (123 ± 2.4; *p* < 0.001), in the Hcy-ISCH group to 301% (61 ± 4.7; *p* < 0.001) and the increase was up to 472% (95.5 ± 4.7; *p* < 0.001) in the Hcy-IR-72h group against the naive control (C). H3K9+ cells in Hcy-ISCH group declined by 50% (*p* < 0.001) and by 78% (*p* < 0.01) in Hcy-IR-72h group when compared to the Hcy-C group.

It can be seen from the micrographs that induction of hHcy significantly affected the histone acetylation status in the M1 cortical region, which is obvious by the increased number H3K9ac+ cells (Figure 2G). Moreover, H3K9ac+ cells between the naive ISCH and Hcy-ISCH group were elevated to 841% (7.25 ± 1.7; *p* < 0.001) and when comparing naive and Hcy-treated IR-72h groups decreased to 66% (*p* < 0.001). Additionally, the elevated H3K9ac immunopositivity in both IR-72h groups was statistically significant in the naive ISCH (*p* < 0.001) and Hcy-ISCH group (*p* < 0.001).

In contrast, the differences in the levels of H4K12ac in cortex between the naive and Hcy-treated groups were less prominent, but still significant. The highest H4K12ac immunopositivity was observed in the naive control (C) and Hcy-C group (Figure 3A–F). Ischemic insult resulted in a significant decline in the number of H4K12ac+ cells to 52% (65 ± 6.4; *p* < 0.001) in the naive ISCH group when compared to the naive control (C). The elevation in immunolabelled H4K12ac cells in the Hcy-C group to 118% (147.5 ± 11; *p* < 0.05) was also statistically significant when compared to the naive control (C). Ischemia and IRI induced significant decline in the number of H4K12+ cell in the naive ISCH group (44%; *p* < 0.001) and in the naive IR-72h to 64% (94 ± 5.2; *p* < 0.01) compared to the Hcy-C group. The decrease by 75% (110 ± 7.2; *p* < 0.05) between the Hcy-C and Hcy-ISCH group was also statistically significant. Furthermore, the increase by 169% (*p* < 0.01) between the Hcy-ISCH and naive ISCH group was significant, as well as the 128% elevation (120 ± 3.9; *p* < 0.05) among the Hcy-IR-72h and naive IR-72h groups. In addition, the difference in H4K12ac+ cells in naive IR-72h group compared to naive IR-72h was significantly elevated, too (Figure 3G).

### 2.3. Detection of H3K9ac and H4K12ac Levels in Cortical Homogenates

**Figure 4:**
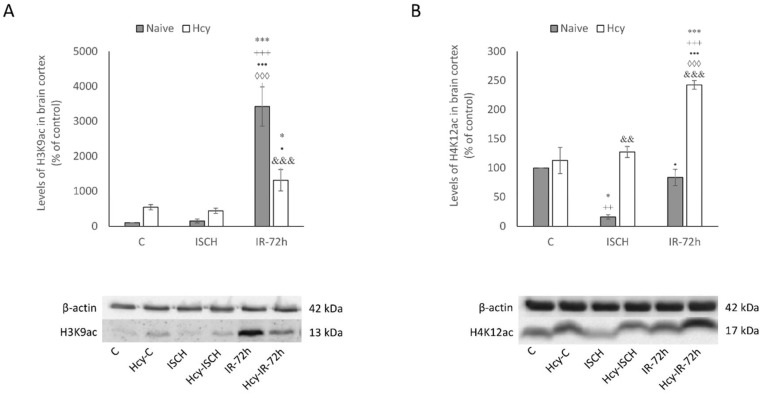
Western blot analysis of H3K9ac (**A**) and H4K12ac (**B**) levels in rat brain cortex homogenates. Levels of histones acetylation in the M1 area in naive control, Hcy-C, naive ISCH, Hcy-ISCH, naive IR-72h and Hcy-IR-72h. Results are presented as mean ± SEM, *n* = 5/group, normalized to the control levels. * *p* < 0.05 and *** *p* < 0.001 versus the control value; ^++^ *p* < 0.01 and ^+++^ *p* < 0.001 versus the control with Hcy; ^•^ *p* < 0.05 and ^•••^ *p* < 0.001 versus the naive ISCH group; ^◊◊◊^ *p* < 0.001 versus the ISCH with Hcy; ^&&^ *p* < 0.01 and ^&&&^ *p* < 0.001 versus the corresponding reperfusion period.

To support the above-mentioned immunohistochemical results, the changes in histone acetylation in the rat brain cortex in all experimental groups were also examined by Western blot analysis. We documented similar differences in histone acetylation in cortical tissue homogenates. Hyperacetylation of H3K9 was the most significant after the IRI in the naive IR-72h group (3422%; *p* < 0.001) when compared to the naive control (C). Surprisingly, the combination of IRI and induced hHcy in the Hcy-IR-72h group resulted in the increase of H3K9ac up to only 1316% (*p* < 0.05) in comparison to naive control (C), which declined markedly by 2.6-times (*p* < 0.001) in comparison to naive IR-72h group (Figure 4A).

Regarding the levels of H4K12ac in cortex, the combination of Hcy treatment and IRI in the Hcy-IR-72h group resulted to a significant elevation by 2.4-times (*p* < 0.001) when compared to the naive control (C). In the naive ISCH group, level of H4K12ac was 6.26-times lower (*p* < 0.05) than the naive control (C) and 7-times lower (*p* < 0.01) than in the Hcy-C group. Remarkably, the elevation of H4K12ac in Hcy-ISCH group almost 8-times (*p* < 0.01) was significant when compared to the naive ISCH group, as the detected level of H4K12ac was nearly 3-times higher between the naive IR-72h and Hcy-IR-72h group (*p* < 0.001). Nevertheless, we observed markedly increased H3K9ac level between the naive control (C) and Hcy-C group, as well as a smaller increase in H4K12ac, none of these changes observed by Western blot were statistically significant (Figure 4B).

Based on our morphological and Western blot observations, we could assume that the combination of IRI with hHcy triggers different responses of H3K9 and H4K12 histone acetylation patterns in the M1 cortical region. This conclusively suggest that hHcy conditions exaggerate processes linked with neuronal damage and epigenetic changes are likely to participate in the mechanism of neurodegeneration induced by IRI and hHcy.

## 3. Discussion

Despite numerous studies devoted to the explanation of stroke etiology, we still have only limited knowledge describing the complex mechanisms of neuronal damage. It is well known that ischemia induces significant changes in the expression of regulatory genes within the biological response to injury, but it also triggers alterations in the epigenetic patterns of the injured organ [35,36,37]. In spite of the known epigenetic influence in the development of various pathological states, only limited studies can be found that describe histone acetylation after IRI under hHcy conditions. Thus, the aim of the present study was to determine how IRI in combination with hHcy affects the extent of neurodegeneration and alters histone acetylation patterns in brain cortex.

Ischemic insult initiates disruption of the blood-brain barrier (BBB) that allows for easier penetration of toxicants such as Hcy [38]. Hcy in turn can induce vulnerable tissue damage, predominantly in the cerebral cortex and hippocampus [31,33]. Moreover, it can cause cellular damage by altering the subtle epigenetic state of all neural cells such as neurons, astrocytes and microglia in vulnerable areas [39].

In this paper, we first focused on the analysis of morphological changes after ischemic insult. Similar to our previous studies [31,32], the most prominent disintegration of neural cells was detected in the naive IR-72h group in the CA1 area of hippocampus and cortical region, and hHcy conditions in the Hcy-IR-72h group exaggerate the extent of this neuronal damage.

Several experimental approaches proved that disarrangement of histone acetylation can contribute to the processes linked with neuronal death. Selective histone acetylation is linked with protection of neurons against oxidative injury by enhanced gene regulation of antioxidant enzymes participating in the neuroprotection [40]. In general, tissue ischemia is associated with the rise in a global amount of DNA- and histone methylation, and this increase correlates with the extension of brain injury [22,23]. However, this effect is tissue and gene specific [41]. Conversely, global hypoacetylation of histones H3 and H4 correlates with ischemic/hypoxic conditions [42,43]. However, some studies observed increased levels of histone acetylation after a preconditioning-tolerance building stimuli [43]. Numerous results of experiments demonstrated a massive decrease in the level of histone acetylation in the ischemic brain persisting up to for 2 weeks after the stroke [44,45]. Interestingly, the histone deacetylase activity does not change during the first 6 h after ischemia and it is upregulated within 7 days after the stroke, which corresponds with the period of the partial recovery of mostly penumbral region after injury [24,26]. Higher level of plasma Hcy interferes with the neuronal metabolism in the brain parenchyma. There is no straightforward evidence for the direct Hcy inclusion in the histone acetylation, it changes the methylation levels and subsequently alters the activity of methyl-binding proteins. This leads to the formation of complexes with histone deacetylases and consequently to the posttranslational histone modifications [14,15,16].

Our paper describes, for the first time, how ischemic insult in combination with hHcy affects the H3 and H4 histone acetylation pattern at specific lysine residues in vulnerable brain area. Generally, as seen from our results, the cortical tissue damage in IR-72h group combined with induced hHcy is linked with the changes in acetylation level of histones and responds to the degree of neuronal damage. Moreover, hHcy condition alone triggers changes at the acetylation process, likely within the context of hypomethylation due to the elevated level of Hcy.

Results of the study of Jin et al. [39] documented global H3K9 hyperacetylation in combination with global hypomethylation in cultured rat astrocytes after Hcy exposure. This leans towards the altered regulation of gene expression following Hcy treatment. Apparently, an epigenetic mechanism might regulate Hcy-related metabolic changes including mitochondrial function. Furthermore, another link between the increased Hcy level and histone acetylation was observed by Xu et al. [46] by the elevated level of H3 acetylation in the presence of its metabolite Hcy-thiolactone.

According to the recent study of Patnala et al. [28], global upregulation of H3K9ac is a consistent feature of activated microglia in the ischemic region. Furthermore, these gene-specific H3K9ac modulations may alter the expression of pro- and anti-inflammatory genes in microglia of ischemized tissue. Similar changes were documented in long-term in vitro Hcy-treated microglia by the reduced pro- and increased anti-inflammatory markers after 72 h of Hcy exposure [47]. Besides, more proinflammatory phenotype was manifested in a hHcy model in mice induced by chronic hHcy diet [48,49].

A global transcriptional repression induced by ischemia is reflected by the alterations of histone H3 and H4 in the injured area [50,51]. This is reasonable, since acetyl-CoA, the fundamental cofactor for histone acetyltransferases (HAT), is the only substrate for acetylation reactions. Its supply is in hypoxic/ischemic conditions depleted due to the inhibition of pyruvate dehydrogenase complex [52,53,54] which might result in the reduced activity of HAT. Moreover, several papers point to the connection between increased oxidative stress, resulting in neurotoxicity and changes in histone modifications/acetylation [55,56,57,58,59]. Oxidative stress causes DNA damage, heterochromatin loss and subsequent aberrant gene expression, inappropriate cell cycle activation and neuronal apoptosis. In the light of these findings there is a possibility that these processes relate to other neurodegenerative diseases with a link to oxidative stress [57]. Hcy acts as an excitatory amino acid that increases unregulated reactive oxygen species (ROS) production and elevates susceptibility of neurons to oxidative stress. The brain possesses fewer antioxidant enzymes than other body tissues and thus these conditions make the brain more vulnerable to oxidative stress and initiate neuronal cell death. Microvasculature and the BBB are also affected by hHcy, which are strictly controlled in the cerebral cortex [60].

Many papers described disarrangement in histone acetylation levels also in the neurodegenerative diseases, such as Alzheimer’s and Parkinson’s disease. Interestingly, the hHcy and ischemic/reperfusion damage are recognized as independent risks in these pathologies. Redistribution of acetylation patterns has also been characterized as age-dependent. Nativio et al. [61] observed increased histone acetylation in aged human controls, but decreased acetylation in comparison with AD patients. Hyperacetylation of H3K9 was also observed in the post-mortem brain of AD patients by Lithner et al. [62]. It has been also pointed out that the global increase in histone H3 acetylation is fundamental in amyloid-related pathogenesis and the formation of AD [62,63]. In addition, Narayan et al. [64] found markedly increased acetylation of H3 and H4 histones along with increased total histone levels in the regions of high pathology in post-mortem AD brains and suggest that any acetylation changes could be due to the changes in the total histone protein levels. Remarkably, the cardiac arrest ischemia is likely to induce genes and proteins connected with AD, contributing to neuronal cell death and neurodegeneration of cerebral tissue [65].

Finally, we could only hypothesize about the effect of how altered acetylation of H3K9 and H4K12 participate in the tissue response in the hHcy IRI animal model. The elevated level of H3K9ac in reperfusion period may be due to the activated expression of genes contributing in the response mechanisms initiated by hHcy and ischemic insult. The elevated acetylation in the hHcy controls may be implicated with known inflammatory responses induced by Hcy [5,7,9]. Changes in acetylation patterns of H3K9 and H4K12 might also be a result of depleted acetyl-CoA required for the acetylation process and the Hcy-induced oxidative stress, followed by the ischemic insult.

In summary, our results contribute to the understanding of how ischemic insult, which is combined with recognized risk factor—hHcy, induces changes in the epigenetic status, especially histone acetylation in the cells of vulnerable brain areas. These changes might modify transcriptionally active or inactive states of chromatin and gene expression as a part of tissue response to the insult. In fact, certain combinations of acetyl and also methyl post-translational histone tail lysine modifications may have an antagonistic or cooperative biological effects [1,2,50,51]. Until now, only sparse information can be found which describe alterations of the whole epigenetic machinery in a complex event such as cerebral ischemic injury combined with hHcy.

## 4. Materials and Methods

### 4.1. Animals and Experimental Design

Animal studies were approved by the ethics committee of Jessenius Faculty of Medicine, Comenius University (19 August 2016, approval code: EK 1647/2015) and by the State Veterinary and Food Department of the Slovak Republic (no. 2857/16-221) in the grant titled “Epigenetic and molecular mechanisms of neuroprotection and ischemic tolerance”. The animals were handled according to the European Union legislation “on the protection of animals used for scientific purposes” (Directive 2010/63/EU). In our study, adult male Wistar rats (Velaz, Prague, Czech Republic) were used, weighing 300–400 g (4–6 months) at the beginning of the experiment with total *n* = 72. Animals were maintained in air-conditioned rooms with sustained standard conditions (22 ± 2 °C and 12 h day/night cycle). Availability of food and water was ad libitum. Global forebrain ischemia was performed by using standard 4-vessel occlusion model developed by Pulsinelli and Brierley [66]. Briefly, the rats were placed in an anesthetic box and anesthetized by inhalation of sevoflurane in a mixture of 33% O_2_/66% N_2_O (4.5% sevoflurane for induction of anesthesia and then 3–3.5% sevoflurane for anesthesia maintenance. Rectal temperature was preserved at 37–38 °C during the surgery with a heating pad. Firstly, both vertebral arteries were irreversibly occluded (day 1) by thermocoagulation through the alar foramina. On the day 2, both common carotids were occluded in duration of 15 min. The anesthetic conditions were maintained as described above. Approximately 2 min before carotid occlusion, the anesthesia was removed and 15 min of ischemia with 72 h of reperfusion followed.

Mydriasis, loss of the righting reflex and paw extension were criteria for forebrain ischemia. The rats which showed no seizures during and after ischemia and those that became unresponsive, lost the righting reflex during carotid occlusion were implied for this experiment. Due to different methods of preparation of biological material for biochemical and histological analysis, 10 animals/experimental group were used (5 animals/method of preparation). After ischemia, animals were sacrificed by decapitation or perfusion (depending on the type of proceeding method) in a mild sevoflurane anesthesia. Brains were dissected and processed immediately. Naive control group of animals proceeded by the same surgical procedure besides carotid occlusion.

### 4.2. Induction of hHcy

Hcy (Sigma-Aldrich, Bratislava, Slovak Republic) was dissolved and buffered as described in our previous paper by Kovalska et al. [33] in concentration of 1.2 μmol/g of body weight). The Hcy injections were administered subcutaneously once a day for 21 days according to Matté et al. [67]. Immediately, six experimental groups were established at day 22. It is well-known that Hcy crosses the BBB and presents a maximal level in the brain 15–60 min after Hcy injection [38,67]. Selected doses of Hcy corresponded to pharmacokinetic parameters, as described in the work of Martins et al. [68]. The level of plasma Hcy in rats reached similar concentrations as referred in previous papers from our laboratory [31,32,33,69].

### 4.3. Experimental Groups of Animals

Animals were divided into 6 groups as follows:

(1) naive control group (C)
(2) hHcy control group (Hcy-C)
(3) naive animals that underwent 15 min ischemia without reperfusion (ISCH)
(4) the animals after 21 days with induced hHcy that underwent 15 min ischemia without reperfusion (Hcy-ISCH)
(5) naive group of animals with 15 min of ischemia followed by 72 h of reperfusion (IR-72h)
(6) the animals after 21 days with induced hHcy that underwent 15 min ischemia and 72 h of reperfusion (Hcy-IR-72h)

### 4.4. Cresyl Violet Staining

The above-mentioned groups (groups 1, 2, 5 and 6) of animals (*n* = 5/group) were anesthetized by inhalation of sevoflurane in a mixture of 33% O_2_/66% N_2_O (4.5% sevoflurane for induction of anesthesia and then 3–3.5% sevoflurane for anesthesia maintenance). Following the anesthesia, the rats underwent a transcardial perfusion with 0.1 mol/L phosphate-buffered saline and 4% paraformaldehyde in 0.1 mol/L PBS [31,70]. After decapitation, the brains were removed from the skull, immersed overnight in the same fixative at 4 °C and placed in 30% sucrose for 24 h. Samples were covered with embedding medium (Killik, Bio Optika, Milano, Italy) and immediately frozen by rapid cooling boost in a cryobar (Shannon Cryotome E, Thermo Scientific, Waltham, MA, USA). Serial coronal 30 µm frozen sections were cut, mounted into Superfrost Plus glass (Thermo Fisher Scientific, Dreieich, Germany) and air-dried. Sections were dehydrated through descending grades of ethanol (95%, 70%, 50%, respectively) and brought to distilled water. The slides were then immersed in cresyl violet staining solution (Lachema, Brno, Czech Republic) for 20 min and washed again in distilled water. After dehydration through the ascending grades of ethanol, the sections were immersed two times for 5 min in xylene solution and coverslipped with entellan. The slides were subsequently examined under the Olympus BX41 light microscope (Olympus, Tokyo, Japan). The counting window for detection of neuronal density presented 1 mm^2^. The number of surviving pyramidal cells was calculated in M1 area of cerebral cortex. Neurons were observed in a double-blind manner and counted on three accidental microscopic fields by two observers.

### 4.5. Detection of Acetylated Histones by Fluorescent Immunohistochemistry

Rabbit polyclonal Anti-acetyl-Histone H3 (Lys9) (Merck Millipore, Darmstadt, Germany, 1:100) and Anti-acetyl-Histone H4 (Lys12) (Merck Millipore, Darmstadt, Germany, 1:100) primary antibodies were used for immunofluorescence. Brain sections from groups 1–6 (see Experimental groups of animals) mounted at Superfrost Plus glass (Thermo Fisher Scientific, Dreieich, Germany) were permeabilized with 0.1% Triton X-100, preblocked with 10% BSA for 1 h. Primary antibodies were applied at 1:100 in the 0.1% Triton X-100 solution with 10% BSA for 24 h at 4 °C, followed by washing with PBS at room temperature. Secondary antibodies were applied at 1:100 for 2 h at room temperature. Detection was performed using Alexa Fluor 488 goat anti-rabbit IgG-conjugated secondary antibodies (1:100, Life Technologies, Carlsbad, CA, USA). The slices were washed again and mounted in Fluoromont-G containing 4′,6-diamidino-2-phenylindole (Southern Biotechnology Associates, Birmingham, AL, USA). We did not find any immunoreactivity in samples without primary antibodies. To minimize the differences between animals, the fluorescent immunohistochemical analysis was accomplished for each time of reperfusion per animal four times. Positive neuronal cells were counted in M1 cortical area by each animal at least in three different sections. The slides were scanned with a confocal laser scanning microscope (Olympus FluoView FV10i, Tokyo, Japan), objective of 10× with zoom up to 40× magnification equipped with filter for Alexa Fluor 488. The images captured with confocal microscope were analyzed with Olympus FluoView FV10-ASW software (version 02.01, Olympus, Tokyo, Japan), Quick Photo Micro software (version 2.3, Promicra, Prague, Czech Republic) and sequentially with Adobe Photoshop CS3 Extended (Adobe System, San Jose, CA, USA).

### 4.6. Tissue Homogenates Preparation and Immunodetection

Brain cortex homogenates from groups 1–6 (see Experimental groups of animals) were prepared as described in our previous paper [31] and immunodetection was assessed according to Pilchová et al. [71]. Membranes were probed with antibodies used for immunofluorescence, rabbit polyclonal Anti-acetyl-Histone H3 (Lys9) (Merck Millipore, Darmstadt, Germany, 1:500) and Anti-acetyl-Histone H4 (Lys12) (Merck Millipore, Darmstadt, Germany, 1:500) with secondary goat anti-rabbit IgG HRP-conjugated antibody (Santa Cruz Biotechnology, Inc., Heidelberg, Germany, 1:5000). Immunoblots were visualized by ECL chemiluminescent substrate (ThermoFisher Scientific, Dreieich, Germany) on Molecular Imager Gel Doc XR System (Bio-Rad, Hercules, CA, USA) and bands of interest were analyzed by Quantity One (Bio-Rad, Hercules, CA, USA). All assays were performed in triplicates.

### 4.7. Quantitative Image Analysis

A total of 270 selected fields of view (average number: 90 fields/staining method and antibody; 3 sections per animal) taken at 40× magnification were analyzed by ImageJ software (National Institute of Health, Bethesda, MD, USA). The counting parameters for cresyl violet, H3K9ac and H4K12ac immunoreactive cells in 3 distinct fields of M1 cortical area of rat brain were elected as follows. The sampling grid size was 0.06 × 0.06 cm and the counting frame size was 0.03 × 0.03 cm. The total number of labelled cells was expressed per mm^2^.

### 4.8. Data Analysis

The values acquired from image analysis were proceeded with GraphPad Prism software (version 6.01 for Windows, La Jolla, CA, USA). Data are presented as the mean ± SEM. The inter-group statistical significance of mean differences was assessed by ANOVA (one-way analysis of variance) followed by a Student-Newman-Keuls test. The means of control (C), hHcy control (Hcy-C), ischemic group (ISCH) and hHcy ischemic group (Hcy-ISCH), IRI group (IR-72h) and hHcy IRI group (Hcy-IR-72h) were compared. The results from Western blot analysis were normalized to naive control which represents 100%. A value of *p* < 0.05 was considered to be statistically significant.

## Abbreviations

BBB	Blood-brain barrier
CA1	Cornu ammonis 1
H3K9ac	Acetylated histone H3 at lysine 9
H4K12ac	Acetylated histone H4 at lysine 12
HAT	Histone acetyltransferase
Hcy	Homocysteine
HDAC	Histone deacetylase
hHcy	Hyperhomocysteinemia
IRI	Ischemia-reperfusion injury
M1	Primary motor cortex
ROS	Reactive oxygen species
SAH	S-adenosylhomocysteine
SAM	S-adenosylmethionine
